# Isolation of Genotype V St. Louis Encephalitis Virus in Florida

**DOI:** 10.3201/eid1504.081094

**Published:** 2009-04

**Authors:** Christy L. Ottendorfer, Jason H. Ambrose, Gregory S. White, Thomas R. Unnasch, Lillian M. Stark

**Affiliations:** University of South Florida, Tampa, Florida, USA (C.L. Ottendorfer, J.H. Ambrose, G.S. White, T.R. Unnasch, L.M. Stark); Florida Department of Health, Tampa (C.L. Ottendorfer, J.H. Ambrose, L.M. Stark); University of Alabama, Birmingham, Alabama, USA (G.S. White)

**Keywords:** Virus, St. Louis encephalitis virus, flavivirus, phylogeny, sentinel chicken, surveillance, dispatch

## Abstract

We isolated and characterized St. Louis encephalitis virus (SLEV) from cloacal swabs of naturally exposed adult sentinel chickens in 2006. Phylogenetic analysis of SLEV strains isolated in Florida indicated that Brazilian SLEV circulated in 1972 and 2006; lineages were VA and VB.

In North America, before the introduction of West Nile virus (WNV; *Flavivirus*, *Flaviviridae*) in 1999, St. Louis encephalitis virus (SLEV; *Flavivirus*, *Flaviviridae*) was the most important agent of epidemic viral encephalitis (*1*). SLEV activity is restricted to the Western Hemisphere and outbreaks have occurred in North America since 1933 (*2*). The recent cocirculation of these closely related flaviviruses has raised the possibility that competitive pressures might alter the transmission cycle of WNV, SLEV, or both (*3*,*4*).

In Florida, periodic SLEV outbreaks since the 1950s led to the formation of an arbovirus surveillance program (*5*), anchored by the Florida Sentinel Chicken Arboviral Surveillance Network (*6*). SLEV is maintained in a mosquito-bird-mosquito cycle; amplification occurs in peridomestic birds and *Culex* spp. mosquitoes (*7*). Chickens are chosen as sentinels because they are susceptible to infection and develop antibodies after exposure (seroconversion) (*8*).

We isolated SLEV from naturally infected adult chickens and compared it with previously isolated strains. The envelope region of viral isolates was analyzed because of its biological importance and high immunogenicity in the host (*9*).

## The Study

In Florida, SLEV transmission is sporadic with periods of low (enzootic) and high (epidemic) activity. SLEV was detected by sentinel chickens every year before introduction of WNV (1988–2007) (Figure). Since 2001, limited SLEV activity has been reported (*10*); SLEV may be in a natural decline, or transmission of WNV may influence SLEV cycles, as has been suggested in California (*4*).

**Figure:**
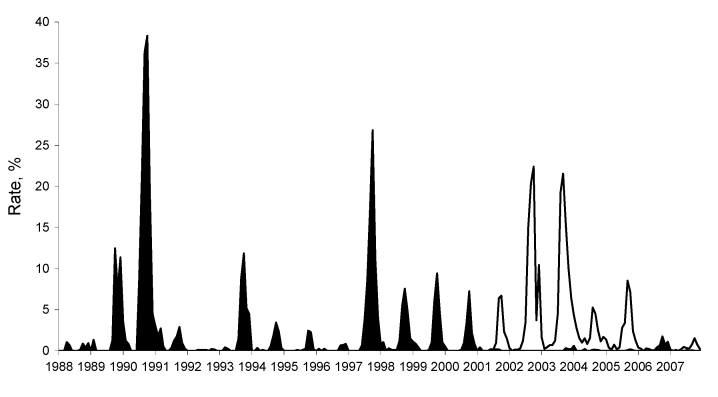
Rates of flavivirus seroconversion in sentinel chickens, Florida, 1988–2007. Black shading shows St. Louis encephalitis virus (SLEV); white shading shows West Nile virus (WNV). Because the number of susceptible sentinel chickens fluctuated during this time, the rates of seroconversion (no. positive chickens/total no. susceptible chickens × 100, per month) are presented rather than numbers of positive birds. SLEV seroconversion rates declined after the 2001 introduction of WNV despite continued surveillance, and an increased number, of susceptible birds located in regions historically at risk for SLEV enzootic transmission.

In 2006, a total of 2,901 adult sentinel chickens were maintained at 275 sites of potential enzootic arbovirus transmission in 34 Florida counties. Blood was collected weekly from each chicken during peak transmission months (July–December) and tested with hemagglutination inhibition assay, immunoglobulin M antibody-capture ELISA, or plaque reduction neutralization test, as previously described (*11*). Sites with confirmed SLEV seroconversions were targeted for sample collection. For the first time since 2001, SLEV sentinel seroconversions (n = 40) exceeded WNV seroconversions (n = 30) (*10*).

In central and south Florida, 5 partner agencies targeted a subset (n = 15) of sentinel chicken sites with recent confirmed arbovirus transmission activity for cloacal swab collection from 95 chickens. During the weekly scheduled bleeding of the flocks, 1,338 cloacal swabs were collected in viral culturettes (Becton Dickinson, Franklin Lakes, NJ, USA); 529 swabs were retrospectively processed for molecular detection assays and virus isolation in Vero cells, as previously described (*12*). Viral RNA was extracted from cloacal swabs and first-passage cell cultures and amplified with real-time reverse transcription–PCR (RT-PCR) TaqMan assays for WNV and SLEV, as previously described (*13*). Two SLEV strains, FL06-S569 and FL06-S650, were detected by RT-PCR and cultured in Vero cells. Fourteen additional SLEV strains were obtained from the Florida Department of Health, Bureau of Laboratories–Tampa archive for phylogenetic analysis (Table).

**Table:** SLEV strains sequenced for phylogenetic analysis*

Strain	Designation	Location	Year	Host	Passage	GenBank accession no.
FL52-Miami	FL52	Miami, FL	1952	Human	SM1, Vero 1	EU906866
TBH-28	TBH-28	Tampa Bay, FL	1962	Human	SM11, Vero 2	EU906867
F72-M022	FL72	Walnut Hill, FL	1972	Opossum	SM3, Vero 1	EU906868
86-100309	FL85a	Indian River, FL†	1985	*Culex nigripalpus* mosquitoes	SM1, Vero 1	EU906869
86-100802	FL85b	Indian River, FL	1985	*C. nigripalpus* mosquitoes	SM2, Vero 1	EU906870
1A-059	FL89	Indian River, FL	1989	Northern cardinal	SM2, Vero 1	EU906871
3-594	FL90a	Indian River, FL	1990	Common grackle	SM1, Vero 1	EU906872
3A-038	FL90b	Indian River, FL	1990	Mourning dove	SM1, Vero 1	EU906873
3-582	FL90c	Indian River, FL	1990	Common grackle	SM1, Vero 1	EU906874
CXN GR8	FL90d	Indian River, FL	1990	*C. nigripalpus* mosquitoes	SM2, Vero 1	EU906875
FL06-S569	FLS569	Sarasota, FL†	2006	Chicken	Vero 1	EU906876
FL06-S650	FLS650	Sarasota, FL	2006	Chicken	Vero 1	EU906877
TRVL21647	TR58	Trinidad	1958	*C. coronator* mosquitoes	SM3, Vero 1	EU906878
TRVL43174	TR62	Trinidad	1962	*C. nigripalpus* mosquitoes	SM4, Vero 1	EU906879
BeAn70092	BR64	Belem, Brazil	1964	Kingfisher	?, SM1, Vero1	EU906880
BeAn156204	BR69	Belem, Brazil	1969	Chicken	SM2, Vero 1	EU906881

*SLEV, St. Louis encephalitis virus; SM, suckling mouse. Twelve strains collected over 5 decades in Florida were sequenced for phylogenetic analysis. Four South American strains of SLEV were acquired by the Bureau of Laboratories–Tampa before 1972 and sequenced as representative of genotype V SLEV. One control strain (TBH-28) was analyzed and used as a positive control in reverse transcription–PCR and sequencing assays. TBH-28 represents a Florida isolate of SLEV made during the 1960s, but the envelope sequence was previously published (GenBank accession no. AF205469). †Indian River and Sarasota counties.

To characterize SLEV strains, we amplified the envelope region using previously described primers (*9*) and the SuperScript III 1-step RT-PCR system (Invitrogen, Carlsbad, CA, USA) following the manufacturer’s instructions. Sequences were aligned by using ClustalW 1.6 and phylogenetic trees drawn by using the maximum parsimony method, with 1,000 bootstrap replicates, in MEGA 4.0 software (*14*), including 60 other SLEV envelope sequences available in GenBank (*9*,*15*) and 4 related flavivirus outgroups (accession nos.: WNV NY99, AF196835; Japanese encephalitis virus, EF571853; Kunjin virus, AY274505; Murray Valley encephalitis virus, AF161266).

The phylogenetic tree places FL06-S569 and FL06-S650 into genotype VA (Appendix Figure). This analysis further supports classification of SLEV into 7 lineages and 13 clades (IA-IB, IIA-IIE, III, IV, VA-VB, VI, VII), as previously suggested (*9*). FL06-S569 and FL06-S650 share 98% sequence identity with SLEV strains from South America, including Brazil (BeAn247377, BeAn242587) and Peru (75D90). Two nucleotide mismatches (silent transition mutations at positions 1083, 1404) were noted in the envelope region within the FL06-S569 and FL06-S650 isolates.

Envelope gene sequences were previously published for 6 Florida strains (*9*), and 9 additional archived Florida isolates were analyzed for this study. Reference strain FL72-M022 was isolated from an opossum from the Florida panhandle in 1972. FL72-M022 shares 97%–98% sequence identity with strains from Brazil (BeAn246262, BeAr23379, and BeH203235) and is placed in genotype VB. In contrast, SLEV reference strains isolated in Florida during 1952 and 1985 share 97%–99% homology with strains collected in Tampa Bay during 1962 (TBH-28, GHA-3) and in Mexico (65V310). The last large outbreak of SLEV in Florida occurred during 1990. Envelope sequence analysis demonstrated that strains isolated during 1989 and 1990 shared 98% homology with USA (V 2380-42), Guatemala (78A28), Tennessee (TNM 4-711), or Texas strains (83V4953, PVI-2419, 98V3181).

## Conclusions

Despite detection of SLEV after the introduction of WNV, SLEV had not recently been cultured by existing statewide surveillance methods in Florida (*10*). Experimental evidence suggests that WNV cross-protective immunity in wild bird species may limit subsequent SLEV infections (*3*). In 2006, sentinel seroconversions supported this hypothesis; limited WNV activity may have enabled increased transmission of SLEV during the fall (Figure).

Partner agencies in the Florida Arbovirus Surveillance Network used a targeted strategy to preferentially sample sentinels located in “hot zones” of SLEV transmission activity for virus isolation and molecular analysis. Sequence analysis of reference strains and the 2006 SLEV isolates has shown the circulation of genotype V SLEV strains in Florida. The 2006 isolates do not represent a recent extension of the geographic range of strains of SLEV from Brazil because 1 genotype V strain was also collected during field studies in 1972. Instead, they support periodic circulation and maintenance of South American SLEV genotypes in Florida, where the diverse ecosystem may allow for evolution of the virus and periodic seeding of SLEV into the United States where the human population may have no immunity to the virus.

On the basis of placement into multiple lineages (IIA-IID, VA, VB) (Appendix Figure), our data support the hypothesis that persistence of SLEV in Florida may differ from its activity in other regions of the United States. For example, the same or highly similar strains of SLEV can be locally maintained for more than a decade in California and Texas (*15*), whereas genetically similar strains of SLEV appear to be infrequently isolated, or maintained at levels below detection, over extended periods in Florida. Our findings suggest periodic introduction of different SLEV genotypes to Florida from the eastern United States and other countries (Mexico, Panama, and Brazil), with distinct North American (lineage II) genotypes isolated in epidemic years. The role of South American genotypes in enzootic or epidemic cycles of SLEV is unknown. In Florida, only the detection of North American genotypes has previously been reported (*9*,*15*), but the isolation of South American strains in 1972 and 2006 suggests a mechanism for the continued maintenance of SLEV. Further isolation and characterization of SLEV strains is needed to improve understanding of the mechanism(s) that favor the amplification of North vs. South American genotypes in Florida.

## Supplementary Material

Appendix Figure Phylogram of the complete envelope region of St. Louis encephalitis virus (SLEV) strains, inferred using the maximum parsimony method in MEGA4 software (*14*). Bootstrap analysis was performed using 1,000 replicates, and the consensus tree (generated by majority rule of 27 most parsimonious trees) was chosen. The number at each node indicates percent branch support by bootstrap sampling; values <50 were collapsed. Branch lengths represent the amount of genetic divergence; the scale bar corresponds to number of base changes in the sequence. The phylogram includes 11 newly sequenced Florida SLEV strains (●), 6 previously sequenced Florida strains (○) (*9*), and 4 newly sequenced South American strains (▲). The phylogram also identified 7 lineages, shown as described in an earlier study of 62 strains (*9*). Florida Genotype V viruses cluster in Lineage VA (FLS569, FLS650) and Lineage VB (FL72).
