# Supplementary figures and images for: Isolation of Genotype V St. Louis Encephalitis Virus in Florida
Source: Emerg Infect Dis. 2009 Apr;15(4):604–6. doi: 10.3201/eid1504.081094 (PMC2671428; doi:10.3201/eid1504.081094)

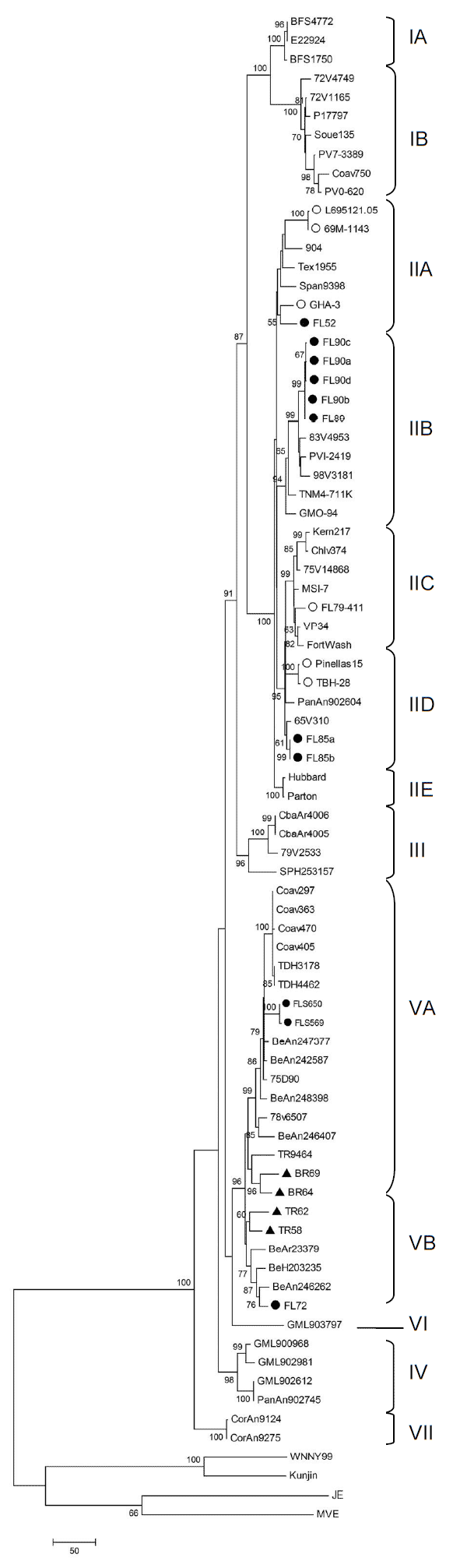

Supplement: Appendix Figure — Phylogram of the complete envelope region of St. Louis encephalitis virus (SLEV) strains, inferred using the maximum parsimony method in MEGA4 software (14). Bootstrap analysis was performed using 1,000 replicates, and the consensus tree (generated by majority rule of 27 most parsimonious trees) was chosen. The number at each node indicates percent branch support by bootstrap sampling; values <50 were collapsed. Branch lengths represent the amount of genetic divergence; the scale bar corresponds to number of base changes in the sequence. The phylogram includes 11 newly sequenced Florida SLEV strains (●), 6 previously sequenced Florida strains (○) (9), and 4 newly sequenced South American strains (▲). The phylogram also identified 7 lineages, shown as described in an earlier study of 62 strains (9). Florida Genotype V viruses cluster in Lineage VA (FLS569, FLS650) and Lineage VB (FL72). [file 08-1094_app-s1.gif]
